# ﻿Ophiostomatalean fungi associated with *Polygraphus* bark beetles in the Qinghai-Tibet Plateau, China

**DOI:** 10.3897/mycokeys.110.135538

**Published:** 2024-11-01

**Authors:** Zheng Wang, Caixia Liu, Xiuyue Song, Yingjie Tie, Huimin Wang, Huixiang Liu, Quan Lu

**Affiliations:** 1 Shandong Research Center for Forestry Harmful Biological Control Engineering and Technology, College of Plant Protection, Shandong Agricultural University, Tai'an 271018, China Shandong Agricultural University Tai'an China; 2 Key Laboratory of Forest Protection of National Forestry and Grassland Administration, Ecology and Nature Conservation Institute, Chinese Academy of Forestry, Beijing 100091, China Ecology and Nature Conservation Institute, Chinese Academy of Forestry Beijing China

**Keywords:** Conifer, forest pest, *Grosmannia*, *Leptographium*, *Ophiostoma*, pine, spruce, symbiosis

## Abstract

Climate change has exacerbated outbreaks of forest pests worldwide. In recent years, bark beetles have caused significant damage to coniferous forests of the Northern Hemisphere. *Polygraphus* bark beetles are widely distributed secondary pests. Recently, tree mortality caused by these beetles on the Qinghai-Tibet Plateau has been increasing; however, few studies have focused on their fungal associations. In the present study, we explored the diversity of ophiostomatalean fungi associated with these beetles on the north-eastern and southern Qinghai-Tibet Plateau. We isolated 442 ophiostomatalean strains from adult beetles and their fresh galleries, specifically targeting *Polygraphuspoligraphus* and *Polygraphusrudis* infesting *Piceacrassifolia* and/or *Pinusgriffithii*. Based on phylogenetic and morphological features, we assigned the 442 strains to 16 species belonging to *Grosmannia* spp., *Leptographium* spp. and *Ophiostoma* spp. Amongst these, *Ophiostomamaixiuense* and *Ophiostomabicolor* were the most frequently isolated species, accounting for 20.8% and 18.1% of the total number of ophiostomatalean assemblages, respectively. By comparing their fungal communities, we found that the different patterns of fungal assemblages of bark beetles from the north-eastern and southern Qinghai-Tibet Plateau may be influenced by biogeographic barriers and host tree species. The results of this study enhance our understanding of bark beetle fungal assemblages, especially *Polygraphus*, on the Qinghai–Tibet Plateau, with implications for forest management under changing climate.

## ﻿Introduction

Extreme heat and frequent droughts driven by climate change have exacerbated forest pest outbreaks (Biedermann et al. 2019). Recently, bark beetles have inflicted severe damage on coniferous forests across the Northern Hemisphere. In Europe, *Ipstypographus* continues to devastate spruce forests, while the frequency of *Ipsacuminatus* outbreaks has increased, leading to significant pine tree mortality (Popkin 2021; Papek et al. 2024). A similar trend has been observed in North America, where elevated temperatures have removed climatic barriers, enabling the northward spread of the aggressive beetles *Dendroctonusfrontalis* and *Dendroctonusponderosae*, which now threaten additional pine forest species and regions (Bentz and Jönsson 2015; Lesk et al. 2017). In China, the Qinghai-Tibet Plateau has not been spared from bark beetle infestations, with species such as *Dendroctonus*, *Ips* and *Polygraphus* causing significant damage (Yin et al. 2016; Wang et al. 2021, 2023). There is growing evidence that fungal symbionts play a crucial role in the ability of bark beetles to respond to climate change and cause tree mortality (Netherer et al. 2021). Despite this, the fungal communities associated with some of these beetles remain poorly understood.

Ophiostomatoid fungi, the most well-known fungal partners of bark beetles, belong to the orders Ophiostomatales (Sordariomycetidae, Sordariomycetes, Ascomycota) and Microascales (Hypocreomycetidae, Sordariomycetes, Ascomycota) (De Beer et al. 2013). Amongst these, the Ophiostomatales is the most diverse group associated with bark beetles, with over 300 species reported across 20 genera (De Beer et al. 2022). The genera *Ophiostoma*, *Leptographium* and *Grosmannia* are particularly notable for their species diversity, close symbiotic relationships with insect vectors and inclusion of species that act as virulent pathogens in host trees. *Ophiostoma* is an ancient genus first described by Sydow and Sydow (1919) and its taxonomy has undergone considerable revision since then. Advances in DNA-based taxonomy and the implementation of the “one fungus, one name” nomenclature have clarified the taxonomic status of this genus. Zipfel et al. (2006) demonstrated that *Ceratocystiopsis* and *Grosmannia* are distinct from *Ophiostoma*, based on multi-gene phylogenies of ribosomal DNA and β-tubulin sequences. Subsequently, *Sporothrix*, which was previously considered part of *Ophiostoma*, was recognised as a separate genus, based on four-gene phylogenies and *sporothrix*-like asexual morphs (De Beer et al. 2016). The taxonomic boundaries between *Grosmannia* and *Leptographium* were historically blurred, but new species in the *Grosmanniapenicillata* complex were later described under the genus *Grosmannia* (De Beer and Wingfield 2013; Yin et al. 2020). Today, these two genera are clearly distinguished, based on genome-wide sequence data (de Beer et al. 2022). Additionally, *Heinzbutinia*, *Jamesreidia* and *Masuyamyces* have been recognised as distinct from *Ophiostoma*. The current taxonomic framework for ophiostomatalean fungi, which is considered the most authoritative, defines *Ophiostoma*, *Leptographium* and *Grosmannia* as comprising six complexes and four groups, eight complexes and two groups and two complexes and one group, respectively (De Beer et al. 2022).

Many ophiostomatoid fungi have been shown to play a positive role in the success of conifer bark beetles, mainly by producing beetle semio-chemicals, exhausting tree defences, providing nutrition and promoting environmental adaptation (Raffa et al. 2015). *Grosmanniapenicillata* and *Leptographiumeurophioides* were found to synthesise the beetle aggregation pheromone 2-methyl-3-buten-2-ol and similar functions have been demonstrated in a variety of ophiostomatoid fungi, indicating their ability to regulate beetle mass attacks (Zhao et al. 2015; Kandasamy et al. 2019, 2023). In contrast, *Endoconidiophorapolonica* can skilfully degrade the phenolic defence compounds of spruce as a carbon source (Wadke et al. 2016), indirectly providing nutrients for its vector, *I.typographus*. Fungal associates of *D.ponderosae*, *Leptographiumclavigerum*, have been shown to contribute to host mortality by triggering the pine tree myriad defence responses (Fortier et al. 2024). Interestingly, the expression of high-altitude adaption-related genes in *Ipsnitidus* was upregulated after feeding on *Ophiostomabicolor*, suggesting that fungal symbionts may promote the adaptation of insect vectors to extreme environments (Wang et al. 2023).

The genus *Polygraphus* is a secondary pest; however, in recent years, it has been reported to cause an increase in tree mortality in Eurasian coniferous forests (Yin et al. 1984; Viklund et al. 2019). This genus is widely distributed in China and mainly attacks conifers, with a few species using hardwoods as a host (Yin et al. 1984). Only a few fungal associates of *Polygraphus* have been reported, most of which have been isolated from mites associated with beetles. Yin et al. (2016, 2019, 2020) successively reported seven ophiostomatalean species associated with *Polygraphuspoligraphus*, three of which were subsequently isolated from beetle mite associates by Chang et al. (2020). In addition, 11 ophiostomatalean species have been isolated from mites associated with *Polygraphusaterrimus*, *P.poligraphus*, *Polygraphusszemaoensis*, *Polygraphusverrucifrons* and *Polygraphus* sp. in Yunnan and Qinghai Provinces (Chang et al. 2017, 2020). Overall, only 18 species from six genera (*Graphilbum*, *Grosmannia*, *Leptographium*, *Masuyamyces*, *Ophiostoma* and *Sporothrix*) associated with five *Polygraphus* beetles were recorded in the two Provinces (Table 1). Although 16 species of this genus have been recorded (Yin et al. 1984; Huang and Lu 2015), most of their fungal associates remain unknown.

**Table 1.** Ophiostomatalean fungi isolated from *Polygraphus* beetles and their mite associates reported from China.

Fungal species	Host	Beetle vector	Location	Reference^1^
*Graphilbumkesiyae*	*Pinuskesiya*	*Polygraphus* sp.; *P.aterrimus*; *P.szemaoensis*	Simao and Ning’er, Yunnan, China	Chang et al. (2017)*
*Gra.puerense*	*P.kesiya*	*P.szemaoensis*	Ning’er Yunnan, China	Chang et al. (2017)*
*Grosmanniacrassifolia*	*Piceacrassifolia*	*P.poligraphus*	Zeku, Qinghai, China	Yin et al. (2020)
*G.maixiuense*	*P.crassifolia*	*P.poligraphus*	Zeku, Qinghai, China	Yin et al. (2020)
*G.xianmiense*	*P.crassifolia*	*P.poligraphus*	Zeku and Menyuan, Qinghai, China	Yin et al. (2020); Chang et al. (2020)*
*Leptographiumbreviscapum*	*P.crassifolia*	*P.poligraphus*	Zeku, Qinghai, China	Yin et al. (2019); Chang et al. (2020)*
*L.conjunctum*	*P.kesiya*	*Polygraphus* sp.	Ning’er Yunnan, China	Chang et al. (2017)*
*L.xiningense*	*P.crassifolia*	*P.poligraphus*	Menyuan, Qinghai, China	Yin et al. (2019)
*L.yunnanense*	*P.kesiya*	*P.szemaoensis*; *Polygraphus* sp.	Ning’er Yunnan, China	Chang et al. (2017)*
*Masuyamycesacarorum*	*P.kesiya*	*P.szemaoensis*	Ning’er Yunnan, China	Chang et al. (2017)*
*Ophiostomaainoae*	*P.crassifolia*	*P.poligraphus*	Zeku, Qinghai, China	Yin et al. (2016); Chang et al. (2020)*
*O.bicolor*	*P.crassifolia*	*P.poligraphus*	Zeku, Qinghai, China	Chang et al. (2020)*
*O.ips*	*P.kesiya*	*P.szemaoensis*; *Polygraphus* sp.	Simao and Ning’er, Yunnan, China	Chang et al. (2017)*
*O.nitidum*	*P.crassifolia*	*P.poligraphus*	Zeku, Qinghai, China	Chang et al. (2020)*
*O.qinghaiense*	*P.crassifolia*	*P.poligraphus*	Zeku, Qinghai, China	Yin et al. (2016)
*O.quercus*	*P.kesiya*; *P.yunnanense*	*P.verrucifrons*; *P.szemaoensis*	Simao and Ning’er, Yunnan, China	Chang et al. (2017)*
*O.shangrilae*	*P.crassifolia*	*P.poligraphus*	Zeku, Qinghai, China	Chang et al. (2020)*
*Sporothrixnebularis*	*P.kesiya*	*Polygraphus* sp.	Ning’er Yunnan, China	Chang et al. (2017)*

^1^ *represents the references on fungal isolation from mites associated with *Polygraphus* beetles.

In the present study, a survey of fungi associated with *P.poligraphus* and *Polygraphusrudis* was conducted on the Qinghai-Tibet Plateau between 2019 and 2020. We sought to increase our understanding of the fungal assemblages associated with *Polygraphus* beetles, based on the accurate identification and comparison of fungal associates across geographic ranges, hosts and beetle vectors.

## ﻿Materials and methods

### ﻿Sample collection and isolation

Adult beetles of *P.poligraphus* and *P.rudis* and/or their galleries were collected during the emergence period from four sites on the north-eastern and southern Qinghai-Tibet Plateau from 2019 to 2020 (Suppl. material 1: table S1). The branches or trunks of the host tree damaged by the beetles were cut into one-metre-long logs and brought back to the laboratory. After peeling the bark, 15 vigorous adults and/or their fresh galleries were selected for fungal isolation from each *Polygraphus* species at each sampling site. Each adult was separated into approximately 15 tissue pieces and transferred to the surface of 2% water agar. The galleries were surface-disinfected with 1.5% sodium hypochlorite and then placed on the surface of 2% water agar. After incubation in the dark at 25 °C, single hyphal tips were transferred to the surface of 2% malt extract agar (MEA) medium to purify the fungal isolates. All strains were deposited in the culture collection at the Forest Pathology Laboratory of the Chinese Academy of Forestry (CXY). Representative strains were deposited at the China Forestry Culture Collection Center, Beijing, China (CFCC).

### ﻿Morphological studies

The morphological structure of each pure culture was carefully observed using an Olympus BX43 microscope (Olympus Corporation, Tokyo, Japan) and recorded using a BioHD-A20c colour digital camera (FluoCa Scientific, China, Shanghai). For the holotype of the new species, we measured the lengths and widths of 30 reproductive structures and presented the following format: (minimum–) mean minus standard deviation−mean plus standard deviation (–maximum). 5-mm diameter agar plugs were transferred from the actively growing margin of fungal colonies and placed in the centre of a 90-mm-diameter Petri plate containing 2% MEA to conduct cultural character studies. Five replicates of culture were incubated at temperatures ranging from 5 °C to 40 °C at 5 °C intervals in darkness. The colony diameters were measured daily until the mycelia reached the margins of the Petri dishes. Culture features were observed and recorded daily until the colonies no longer showed any significant changes. All the data from the type specimens were deposited in MycoBank (www.MycoBank.org).

### ﻿DNA extraction, PCR amplification and sequencing

Actively growing mycelia of each representative strain were collected for DNA extraction using an Invisorb Spin Plant Mini Kit (Tiangen, Beijing, China), following the manufacturer’s instructions. The internal transcribed spacer regions 1 and 2 of the nuclear ribosomal DNA operon, including the 5.8S region (ITS), internal transcribed spacer 2, part of the 28S of the rDNA operon (ITS2-LSU), β-tubulin gene region (*tub2*) and transcription elongation factor 1-α gene region (*tef1-α*) were amplified using the primer pairs of ITS1-F/ITS4 (White et al. 1990; Gardes and Bruns 1993), ITS3/LR3 (Vilgalys and Hester 1990; White et al. 1990), Bt2a/Bt2b (Glass and Donaldson 1995) or T10/Bt2b (O’Donnell and Cigelnik 1997) or EF1F/EF2R (Jacobs et al. 2004), respectively, using 2 × Taq PCR MasterMix (Tiangen, Beijing, China), following the manufacturer’s instructions. PCR and sequencing were performed according to protocols described by Wang et al. (2020, 2021).

### ﻿Phylogenetic analysis

Newly-obtained sequences were identified using a standard nucleotide BLAST search in NCBI and deposited in GenBank. Reference sequences in the phylogenetic analyses were confirmed, based on the BLAST results, relevant literature and sequences downloaded from GenBank. MAFFT v.7 (Katoh et al. 2019) was used to construct the multiple sequence alignment. Molecular Evolutionary Genetic Analyses (MEGA) 7.0 (Kumar et al. 2016) were used to edit and/or splice alignments to generate combined gene datasets.

Maximum Likelihood (ML) analyses were conducted using RAxML-HPC v.8.2.3 (Stamatakis 2014) with 1000 replicates using the GTRGAMMA model. The bootstrap support values of the nodes were estimated using 1,000 bootstrap replicates after retaining the best tree. jModelTest v.2.1.7 (Darriba et al. 2012) was used to determine the best substitution models for conducting Bayesian Inference (BI) analyses in MrBayes v. 3.1.2 (Ronquist and Huelsenbeck 2003). Four Markov Chain Monte Carlo (MCMC) chains were run simultaneously from a random starting tree for 10,000,000 generations. The trees were sampled every 100 generations. Posterior probabilities were calculated, based on the remaining trees after discarding the first 25% of the sampled trees. Phylogenetic trees were edited and polished using FigTree v.1.4.3 (http://tree.bio.ed.ac.uk/software/figtree/) and Adobe Illustrator CS6. The final sequence datasets were submitted to TreeBASE (31618).

## ﻿Results

### ﻿Sampling collection and fungal isolation

In the present study, 442 ophiostomatalean strains were isolated from 75 vigorous adult *Polygraphus* species and 180 fresh galleries of *Piceacrassifolia* and *Pinusgriffithii*. Morphological characterisations and *tub2* or ITS sequence features, based on standard nucleotide BLAST searches at GenBank, were used for preliminary identification. Subsequently, 49 representative strains were selected for detailed morphological and phylogenetic analyses (Table 2).

**Table 2.** Representative strains of ophiostomatalean fungi isolated from *Polygraphus* bark beetles in this study. ^1^CFCC: the China Forestry Culture Collection Center; CXY: the culture collection at the Forest Pathology Laboratory of the Chinese Academy of Forestry.

Species	Taxon	Isolate no^1^	Host	Insect vector	Location	GenBank accession no		
						ITS or ITS2-LSU	tub2	tef1-α
*Grosmannia*								
*G.penicillata* complex								
*G.crassifolia*	1	CFCC57904	*Piceacrassifolia*	*Polygraphuspoligraphus*	Zeku, Qinghai, China	PQ166546	PQ166449	PQ166498
*G.maixiuensis*	2	CFCC57902	*P.crassifolia*	*P.poligraphus*	Zeku, Qinghai, China	PQ166547	PQ166450	PQ166499
		CFCC57903	*P.crassifolia*	*P.poligraphus*	Zeku, Qinghai, China	-	PQ166451	PQ166500
*Grosmannia* sp. 1	3	CFCC57905	*P.crassifolia*	*P.rudis*	Zeku, Qinghai, China	PQ166548	PQ166452	PQ166501
		CFCC57906	*P.crassifolia*	*P.rudis*	Zeku, Qinghai, China	-	PQ166453	PQ166502
		CFCC57907	*P.crassifolia*	*P.poligraphus*	Qilian, Qinghai, China	-	PQ166454	PQ166503
		CFCC57908	*P.crassifolia*	*P.poligraphus*	Qilian, Qinghai, China	-	PQ166455	PQ166504
*Leptographium*								
*L.lundbergii* complex								
*L.griffithii*	4	CFCC57893	*Pinusgriffithii*	*P.rudis*	Yadong, Tibet, China	PQ166549	PQ166456	PQ166505
		CFCC57894	*P.griffithii*	*P.rudis*	Yadong, Tibet, China	-	PQ166457	PQ166506
		CFCC57895	*P.griffithii*	*P.rudis*	Yadong, Tibet, China	-	PQ166458	PQ166507
*L.jilongense*	5	CFCC57896	*P.griffithii*	*P.rudis*	Jilong, Tibet, China	PQ166550	PQ166459	PQ166508
*L.pseudojilongense*	6	CFCC57901	*P.griffithii*	*P.rudis*	Jilong, Tibet, China	PQ166551	PQ166460	PQ166509
		CXY3348	*P.griffithii*	*P.rudis*	Jilong, Tibet, China	-	PQ166461	PQ166510
		CXY3349	*P.griffithii*	*P.rudis*	Jilong, Tibet, China	-	PQ166462	PQ166511
*L.yadongense*	7	CFCC57897	*P.griffithii*	*P.rudis*	Yadong, Tibet, China	PQ166552	PQ166463	PQ166512
		CFCC57898	*P.griffithii*	*P.rudis*	Yadong, Tibet, China	-	PQ166464	PQ166513
		CFCC57899	*P.griffithii*	*P.rudis*	Yadong, Tibet, China	-	PQ166465	PQ166514
		CFCC57900	*P.griffithii*	*P.rudis*	Yadong, Tibet, China	-	PQ166466	PQ166515
*L.olivaceum* complex								
*L.breviscapum*	8	CFCC57890	*P.crassifolia*	*P.poligraphus*	Zeku, Qinghai, China	PQ166553	PQ166467	PQ166516
		CFCC57891	*P.crassifolia*	*P.poligraphus*	Zeku, Qinghai, China	-	PQ166468	PQ166517
		CFCC57892	*P.crassifolia*	*P.poligraphus*	Zeku, Qinghai, China	-	PQ166469	PQ166518
*Ophiostoma*								
*O.clavatum* complex								
*O.pseudobrevipilosi*	9	CFCC57916	*P.griffithii*	*P.rudis*	Yadong, Tibet, China	-	PQ166470	-
		CFCC57917	*P.griffithii*	*P.rudis*	Yadong, Tibet, China	PQ166530	PQ166471	-
		CFCC57918	*P.griffithii*	*P.rudis*	Yadong, Tibet, China	-	PQ166472	-
		CFCC57919	*P.griffithii*	*P.rudis*	Yadong, Tibet, China	-	PQ166473	-
*O.stebbingi*	10	CFCC57920	*P.griffithii*	*P.rudis*	Jilong, Tibet, China	-	PQ166474	PQ166519
		CFCC57921	*P.griffithii*	*P.rudis*	Jilong, Tibet, China	PQ166531	PQ166475	-
		CFCC57922	*P.griffithii*	*P.rudis*	Jilong, Tibet, China	-	PQ166476	-
*Ophiostoma* sp. 1	11	CFCC57923	*P.griffithii*	*P.rudis*	Jilong, Tibet, China	PQ166532	PQ166477	PQ166520
		CFCC57924	*P.griffithii*	*P.rudis*	Jilong, Tibet, China	-	PQ166478	-
		CFCC57925	*P.griffithii*	*P.rudis*	Jilong, Tibet, China	-	PQ166479	-
*O.ips* complex								
*O.bicolor*	12	CFCC57909	*P.crassifolia*	*P.poligraphus*	Zeku, Qinghai, China	PQ166533	PQ166480	-
		CFCC57910	*P.crassifolia*	*P.poligraphus*	Zeku, Qinghai, China	-	PQ166481	-
		CFCC57911	*P.crassifolia*	*P.poligraphus*	Zeku, Qinghai, China	-	PQ166482	-
		CFCC57912	*P.crassifolia*	*P.poligraphus*	Qilian, Qinghai, China	-	PQ166483	-
*O.shigatsense*	13	CFCC57913	*P.griffithii*	*P.rudis*	Jilong, Tibet, China	PQ166534	PQ166484	-
		CFCC57914	*P.griffithii*	*P.rudis*	Jilong, Tibet, China	-	PQ166485	-
		CFCC57915	*P.griffithii*	*P.rudis*	Jilong, Tibet, China	-	PQ166486	-
Group A								
*O.maixiuense*	14	CFCC57930	*P.griffithii*	*P.rudis*	Jilong, Tibet, China	PQ166535	PQ166487	-
		CFCC57931	*P.griffithii*	*P.rudis*	Jilong, Tibet, China	PQ166536	PQ166488	-
		CFCC57932	*P.crassifolia*	*P.poligraphus*	Zeku, Qinghai, China	PQ166537	PQ166489	-
		CFCC57933	*P.crassifolia*	*P.poligraphus*	Zeku, Qinghai, China	PQ166538	PQ166490	-
		CFCC57934	*P.griffithii*	*P.rudis*	Yadong, Tibet, China	PQ166539	PQ166491	-
		CFCC57935	*P.griffithii*	*P.rudis*	Yadong, Tibet, China	PQ166540	PQ166492	-
*O.pacis*	15	CFCC57936	*P.crassifolia*	*P.poligraphus*	Zeku, Qinghai, China	PQ166541	PQ166493	-
*O.sanum*	16	CFCC57926	*P.crassifolia*	*P.rudis*	Zeku, Qinghai, China	PQ166542	PQ166494	-
		CFCC57927	*P.crassifolia*	*P.rudis*	Zeku, Qinghai, China	PQ166543	PQ166495	-
		CFCC57928	*P.crassifolia*	*P.rudis*	Zeku, Qinghai, China	PQ166544	PQ166496	-
		CFCC57929	*P.crassifolia*	*P.rudis*	Zeku, Qinghai, China	PQ166545	PQ166497	-

### ﻿Phylogenetic analysis

#### ﻿*Grosmannia* spp. and *Leptographium* spp.

**Figure 1.**
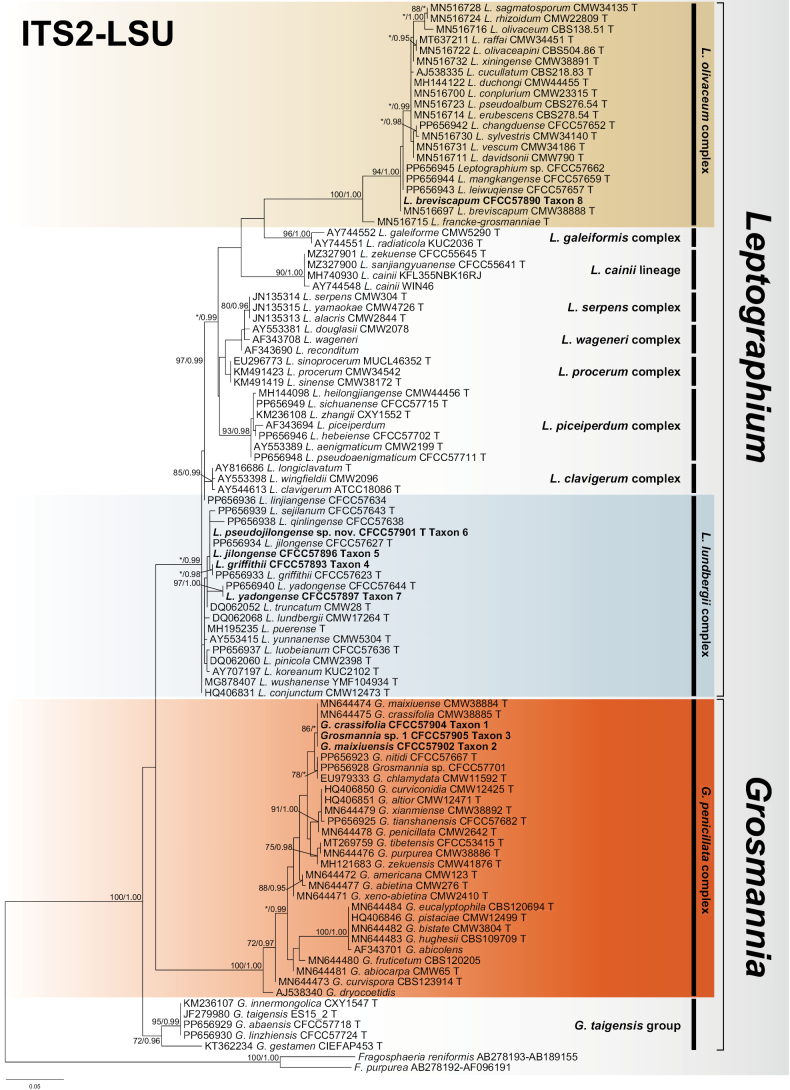
Phylogram of *Grosmannia* spp. and *Leptographium* spp. based on ITS2-LSU sequence data. The ML bootstrap support values ≥ 70% and posterior probability values ≥ 0.9 are recorded at the nodes. T = ex-type isolates.

The ITS2-LSU dataset was used to construct phylogenetic inferences for the two genera. The dataset contained 610 characters, including gaps and the best evolutionary model for BI analysis was estimated to be GTR+I+G. The results showed that our eight representative isolates nested into three complexes, namely the *G.penicillata*, *L.lundbergii* and *L.olivaceum* complexes (Fig. 1). Amongst these, the *G.penicillata* complex belongs to *Grosmannia*, whereas the *L.lundbergii* and *L.olivaceum* complexes belong to *Leptographium*. Subsequently, we constructed the phylogenetic inference of *tub2*, *tef1-α* and the concatenated (*tub2*+*tef1-α*) datasets for each complex.

#### ﻿*Grosmanniapenicillata* complex

**Figure 2.**
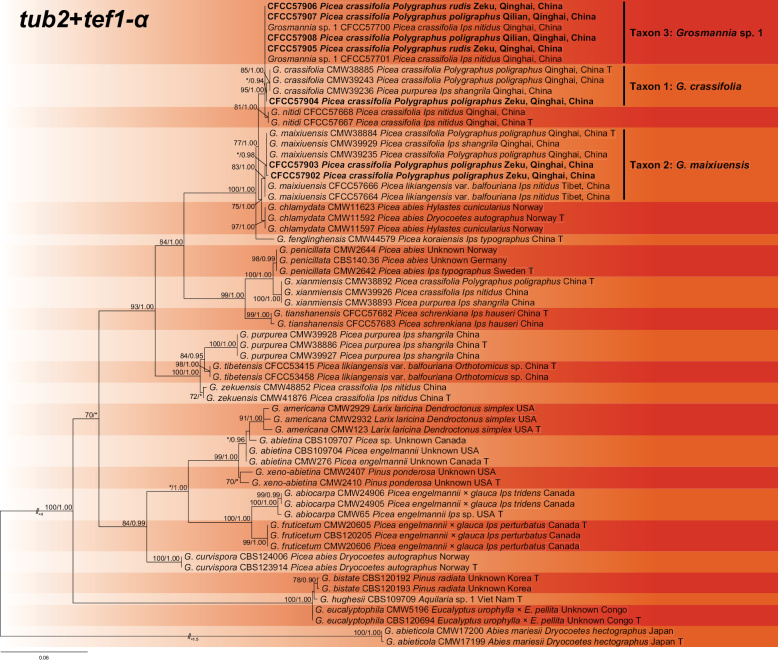
Phylogram of *Grosmanniapenicillata* complex (including Taxa 1–3) based on combined (*tub2*+*tef1-α*) sequence data. The ML bootstrap support values ≥ 70% and posterior probability values ≥ 0.9 are recorded at the nodes. T = ex-type isolates.

The *tub2*, *tef1-α* and concatenated (*tub2*+*tef1-α*) datasets were aligned (containing 402, 694 and 1096 characters, including gaps, respectively) and used to construct the phylogenetic inference. The best models of the three datasets for BI analysis were estimated as HKY+I (*tub2* dataset) and GTR+G (*tef1-α* and concatenated datasets). Based on the concatenated tree (Fig. 2), the seven isolates formed three separate well-supported terminal clades, representing two known and one undescribed taxa: *G.crassifolia* (Taxon 1), *G.maixiuensis* (Taxon 2) and *Grosmannia* sp. 1 (Taxon 3). These three species formed a subclade with *G.chlamydata* and *G.nitidi* that was phylogenically consistent, based on the three datasets (Fig. 2, Suppl. material 2: figs S1, S2).

#### ﻿*Leptographiumlundbergii* complex

**Figure 3.**
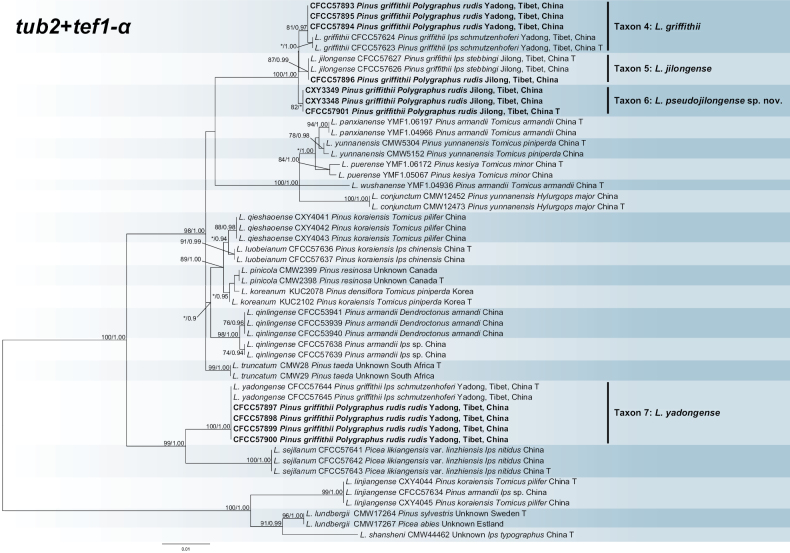
Phylogram of *Leptographiumlundbergii* complex (including Taxa 4–7) based on combined (*tub2*+*tef1-α*) sequence data. The ML bootstrap support values ≥ 70% and posterior probability values ≥ 0.9 are recorded at the nodes. T = ex-type isolates.

The *tub2*, *tef1-α* and concatenated (*tub2*+*tef1-α*) datasets were aligned (containing 373, 666 and 1039 characters, including gaps, respectively) and used to construct the phylogenetic inference. The best models of the three datasets for BI analysis were SYM+I, HKY+G and GTR+G. Based on the concatenated tree (Fig. 3), our ten isolates formed four separate well-supported terminal clades, representing three known (Taxon 4: *L.griffithii*; Taxon 5: *L.jilongense*; Taxon 7: *L.yadongense*) and one undescribed (Taxon 6) taxa. Taxa 4, 5 and 6 were sister species and formed a subclade with *L.panxianense*, *L.yunnanense*, *L.puerense*, *L.wushanense* and *L.conjunctum*, all of which were isolated from *Pinus* trees in southwest China (Fig. 3, Suppl. material 2: figs S3, S4). The four isolated strains were identical in sequence to the two strains isolated from *Ipsschmutzenhoferi*, representing *L.yadongense*, which was a phylogenetic sister to *L.sejilanum* (Fig. 3, Suppl. material 2: figs S3, S4).

#### ﻿*Leptographiumolivaceum* complex

**Figure 4.**
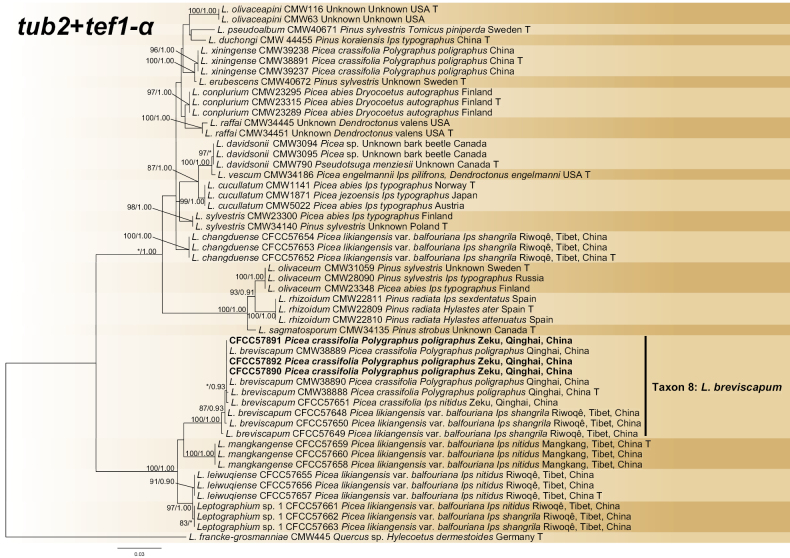
Phylogram of *Leptographiumolivaceum* complex (including Taxon 8) based on combined (*tub2*+*tef1-α*) sequence data. The ML bootstrap support values ≥ 70% and posterior probability values ≥ 0.9 are recorded at the nodes. T = ex-type isolates.

The *tub2*, *tef1-α* and concatenated (*tub2*+*tef1-α*) datasets were aligned (containing 278, 677 and 955 characters, including gaps, respectively) and used to construct the phylogenetic inference. The best models of the three datasets for BI analysis were estimated as (*tub2* dataset) and GTR+G (*tef1-α* and concatenated datasets). Based on the concatenated tree (Fig. 4), the three isolates formed a separate, well-supported, terminal clade representing *L.breviscapum* (Taxon 8). The 10 strains of *L.breviscapum* formed a subclade with *L.leiwuqiense*, *L.mangkangense* and *Leptographium* sp. 1, all of which were isolated from *Picea* trees on the Qinghai-Tibet Plateau (Fig. 4, Suppl. material 2: figs S5, S6).

#### ﻿*Ophiostoma* spp.

**Figure 5.**
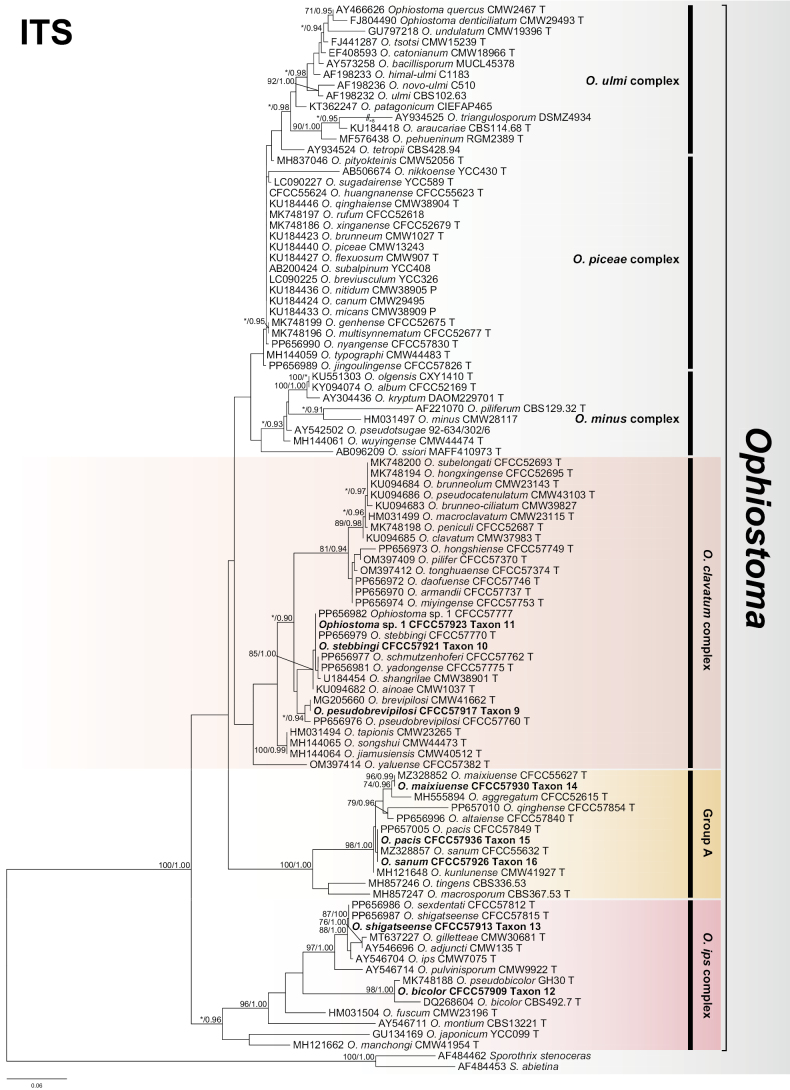
Phylogram of *Ophiostoma* spp. based on ITS sequence data. The ML bootstrap support values ≥ 70% and posterior probability values ≥ 0.9 are recorded at the nodes. T = ex-type isolates.

An ITS dataset was used to construct a phylogenetic inference for this genus. The dataset contained 743 characters, including gaps and the best evolutionary model for BI analysis was estimated to be GTR+I+G. The results showed that our eight representative isolates nested into two complexes and one Group A, namely the *O.clavatum* complex, *O.ips* complex and Group A (Fig. 5). Subsequently, we constructed the phylogenetic inference of *tub2*, *tef1-α* and/or the concatenated (*tub2+tef1-α* or ITS+*tub2*) datasets for each complex or Group.

#### ﻿*Ophiostomaclavatum* complex

**Figure 6.**
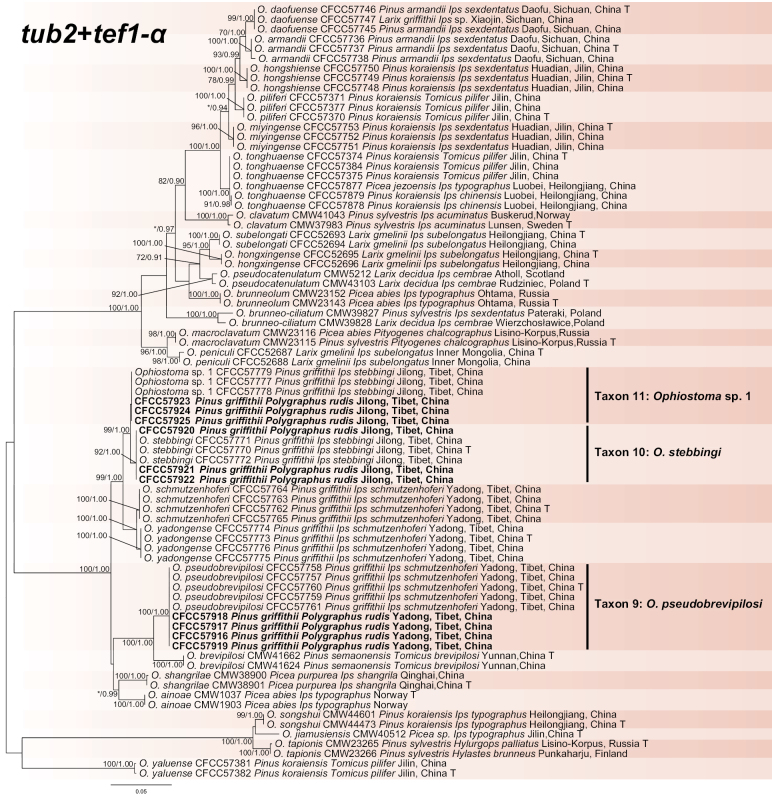
Phylogram of *Ophiostomaclavatum* complex (including Taxa 9–11) based on combined (*tub2*+*tef1-α*) sequence data. The ML bootstrap support values ≥ 70% and posterior probability values ≥ 0.9 are recorded at the nodes. T = ex-type isolates.

The *tub2*, *tef1-α* and concatenated (*tub2*+*tef1-α*) datasets were aligned (containing 438, 594 and 1032 characters, including gaps, respectively) and used to construct the phylogenetic inference. The best models of the three datasets for BI analysis were estimated as HKY+I, GTR+G and GTR+I+G. Based on the concatenated tree (Fig. 6), our ten isolates formed three separate well-supported terminal clades, representing two known (Taxon 9: *O.pseudobrevipilosi*; Taxon 10: *O.stebbingi*) and one undescribed (Taxon 11: *Ophiostoma* sp. 1) taxa. *Ophiostomapseudobrevipilosi*, *O.stebbingi* and *Ophiostoma* sp. 1 formed the main subclade in this complex with *O.ainoae*, *O.brevipilosi*, *O.pseudobrevipilosi*, *O.schmutzenhoferi*, *O.shangrilae* and *O.yadongense* (Fig. 6, Suppl. material 2: figs S7, S8).

#### ﻿*Ophiostomaips* complex

**Figure 7.**
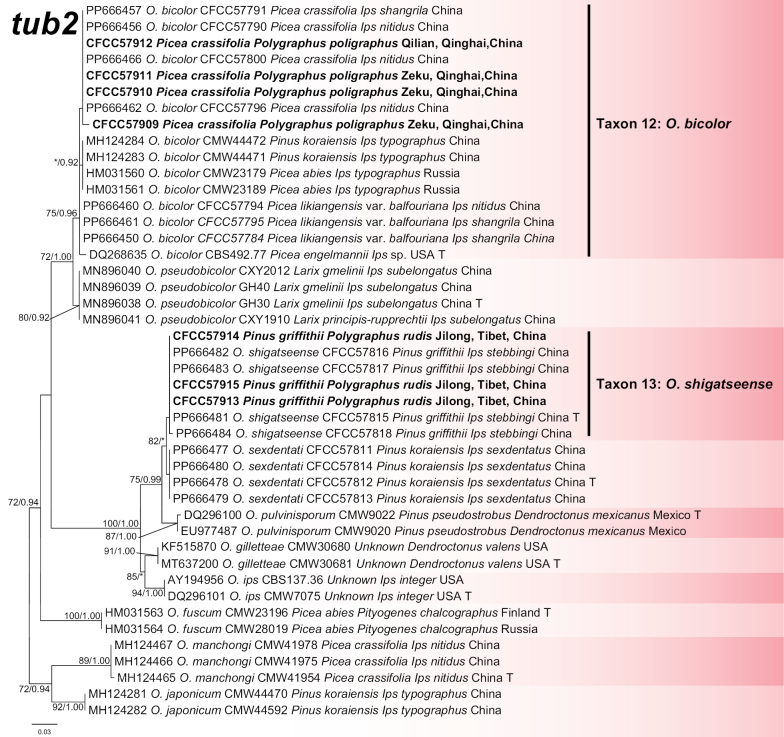
Phylogram of *Ophiostomaips* complex (including Taxa 12–13) based on *tub2* sequence data. The ML bootstrap support values ≥ 70% and posterior probability values ≥ 0.9 are recorded at the nodes. T = ex-type isolates.

The *tub2* dataset was aligned (containing 274 characters including gaps) and used to construct a phylogenetic inference. The best model of the three datasets for BI analysis was estimated to be HKY+I. The seven isolates formed two clades: *O.bicolor* (Taxon12) and *O.shigatsense* (Taxon 13) (Fig. 7).

#### ﻿Group A

**Figure 8.**
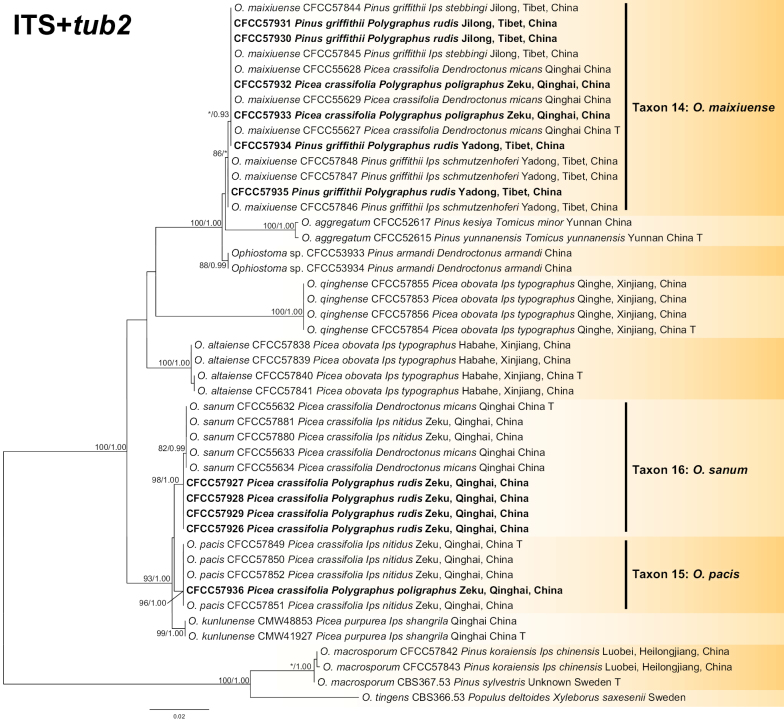
Phylogram of Group A (including Taxa 14–16) based on combined (ITS+*tub2*) sequence data. The ML bootstrap support values ≥ 70% and posterior probability values ≥ 0.9 are recorded at the nodes. T = ex-type isolates.

The ITS, *tub2* and concatenated (ITS+*tub2*) datasets were aligned (containing 685, 445 and 1130 characters, including gaps) and used to construct the phylogenetic inference. The best models of the three datasets for BI analysis were estimated to be GTR+I+G (ITS dataset) and GTR+G (ITS and concatenated datasets). Based on the concatenated tree (Fig. 8), the 11 isolates formed three separate well-supported terminal clades representing three known taxa (Taxon 14: *O.maixiuense*, Taxon 15: *O.pacis* and Taxon 16: *O.sanum*). *Ophiostomamaixiuense* and *O.sanum* showed intraspecific sequence variation and were phylogenetic sisters to *O.aggregatum* and *O.pacis* (Fig. 8, Suppl. material 2: figs S9, S10).

### ﻿Taxonomy

#### ﻿ Leptographium pseudojilongense

Taxon classificationFungiOphiostomatalesOphiostomataceae

Z. Wang & Q. Lu sp. nov.

7F5F5F3A-39B0-574C-846D-86B4A5D0F36E

 855413

Taxon 6, Fig. 9

##### Etymology.

The epithet *pseudojilongense* (Latin) refers to its sister species *L.jilongense*.

##### Holotype.

CXY3312.

##### Description.

**Sexual morph**: not observed. **Asexual morph: *Leptographium***-like. ***Conidiophores*** occurring singly, upright, arising directly from the mycelium, macronematous, mononematous, (247.7–)343.3–484.6(–513.7) μm in length including the conidiogenous apparatus, rhizoid-like structures absent. ***Stipes*** light olivaceous, not constricted, cylindrical, simple, 3–10-septate, (98.8–)103.0–230.0(–301.2) μm in length, (9.0–)11.1–16.9(–18.6) μm wide at base, the basal cell swollen or not, (7.1–)8.5–14.2(–16.6) μm wide below primary branches, apical cell not swollen. ***Conidiogenous apparatus*** (100.8–)180.1–362.1(–417.4) μm in length, excluding the conidial mass, consisting of 1–4 series of branches, the primary branching type B. ***Primary branches*** light olivaceous, cylindrical, (15.4–)19.6–31.8(–35.4) × (6.2–)7.3–10.6(–12.3) μm; ***secondary branches*** light olivaceous, aseptate, (12.4–)13.3–16.7(–18.4) × (6.0–)6.4–9.5(–10.2) μm; ***tertiary branches*** light olivaceous or hyaline, aseptate, (8.0–)8.4–14.0(–16.1) × 5.3–7.6(–8.9) μm. ***Conidiogenous cells*** discrete, 2–3 per branch, smooth or rough, cylindrical, (16.9–)22.2–35.4(–52.6) × (3.9–)4.0–4.8(–5.1) μm. ***Conidia*** hyaline, smooth, aseptate, obovoid, (11.9–)12.9–15.7(–17.9) × (5.5–)6.3–7.8(–8.2) μm.

##### Culture characters.

Colonies on 2% MEA at 25 °C reaching a diameter of 50.1 mm in 4 days, initially hyaline or light white, later becoming light olivaceous from the centre of the colony to the sides, then becoming dark olivaceous, mycelium submerged and superficial with abundant aerial mycelia and the colony margin thinning radially. Optimal temperature for growth was 25 °C, with slow growth observed at 5 °C (45.3 mm in 30 days) and no growth at 35 °C.

##### Associated insects.

*Polygraphusrudis*.

##### Hosts.

*Pinusgriffithii*.

##### Material examined.

China • Xizang Autonomous Region, Shigatse City, Jilong County, from *Polygraphusrudis* infesting *Pinusgriffithii*, July 2019, Z. Wang and Q. Lu, holotype: CXY3312, ex-type culture CFCC57901, ibid. CXY3348, CXY3349.

##### Notes.

**Figure 9.**
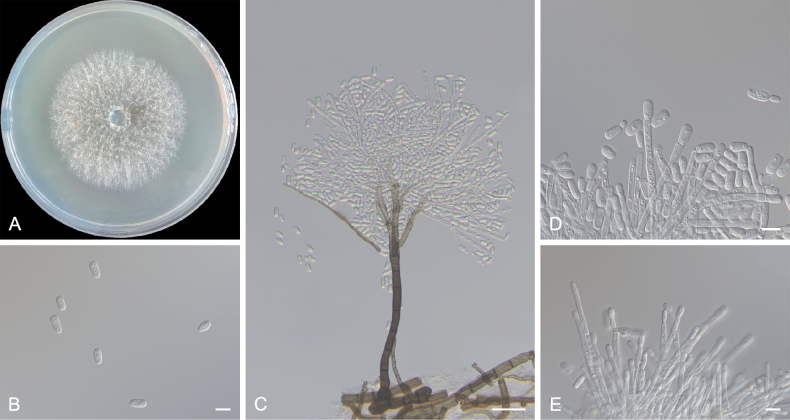
Morphological characteristics of *Leptographiumpseudojilongense* sp. nov. (Taxon 9, CXY3312, holotype) **A** four-day-old cultures on 2% MEA **B–E***Leptographium*-like asexual morph: conidiogenous cells and conidia. Scale bars: 10 μm (**B, D, E**); 40 μm (**C**).

*Leptographiumpseudojilongense* was a phylogenetic sister to *L.griffithii* and *L.jilongense* (Fig. 9), both of which were associated with *Pinusgriffithii* in Shigatse, Xizang (Wang et al. 2024). *Leptographiumpseudojilongense* can be distinguished from *L.griffithii* in the concatenated alignment by 1/373 bp in *tub2* and 3/666 bp in *tef1-α* and from *L.jilongense* in the concatenated alignment by 3/666 bp in *tef1-α*. In terms of morphological characteristics, *L.pseudojilongense* can be distinguished from the other two species by the presence of a *leptographium*-like asexual state, which is absent in the latter two. For culture characteristics, the optimum growth temperature for both was 25 °C, but the former grew slower than the latter two (4 days: 50.1 mm vs. 64.5 and 76.0 mm). At 5 °C, *L.pseudojilongense* was observed growing slowly with 45.3 mm in 30 days, whereas the other two did not grow. Furthermore, *L.pseudojilongense* was isolated from Jilong County, whereas *L.griffithii* and *L.jilongense* were isolated from *Ipsschmutzenhoferi* from Yadong County and *Ipsstebbingi* from Jilong County, respectively.

## ﻿Discussion

In total, 442 ophiostomatalean strains representing 16 species were obtained from adult *Polygraphus* beetles and their galleries in *Piceacrassifolia* and *Pinusgriffithii* on the north-eastern and southern Qinghai-Tibet Plateau. These species were assigned to *Grosmannia* (*G.crassifolia*, *G.maixiuensis* and *Grosmannia* sp. 1 in *G.penicillata* complex), *Leptographium* (*L.griffithii*, *L.jilongense*, *L.pseudojilongense* and *L.yadongense* in *L.lundbergii* complex; *L.breviscapum* in *L.olivaceum* complex) and *Ophiostoma* (*O.pseudobrevipilosi*, *O.stebbingi* and *Ophiostoma* sp. 1 in *O.clavatum* complex; *O.bicolor* and *O.shigatsense* in *O.ips* complex; *O.maixiuense*, *O.pacis* and *O.sanum* in Group A). Amongst them, 12 species were first recorded as associated with *Polygraphus* beetles in China. Yin et al. (2016, 2019, 2020) reported seven ophiostomatalean associates of *P.poligraphus*, but we only collected three of them, which may be because of sample size and sampling time or because the remaining four species are occasional (the previous reports did not count the proportions of each species). To date, three genera (20 species) of ophiostomatalean fungi have been reported to be associated with *Polygraphus* beetles in China (Yin et al. 2016, 2019, 2020), increasing to six genera (30 species) when the fungi isolated from mites associated with these beetles are included (Chang et al. 2017, 2020), showing an abundance of species diversity (Tables 1, 3).

**Table 3.** Strains of ophiostomatalean fungi associated with *Polygraphus* in this study.

Taxon	Genus	Species group and complex	Species	Numbers of isolates^1^					Total	Total Percentages
				PrJ	PrY	PrZ	PpZ	PpQ		
1	*Grosmannia*	*G.penicillata*	*G.crassifolia*				8		8	1.81%
2			*G.maixiuensis*				9		9	2.04%
3			*Grosmannia* sp. 1			10		2	12	2.71%
4	*Leptographium*	*L.lundbergii*	*L.griffithii*		14				14	3.17%
5			*L.jilongense*	1					1	0.23%
6			*L.pseudojilongense*	3					3	0.68%
7			*L.yadongense*		77				77	17.42%
8		*L.olivaceum*	*L.breviscapum*				33		33	7.47%
9	*Ophiostoma*	*O.clavatum*	*O.pseudobrevipilosi*		59				59	13.35%
10			*O.stebbingi*	16					16	3.62%
11			*Ophiostoma* sp. 1	18					18	4.07%
12		*O.ips*	*O.bicolor*				20	60	80	18.10%
13			*O.shigatsense*	4					4	0.90%
14		Group A	*O.maixiuense*	28	39		25		92	20.81%
15			*O.pacis*				1		1	0.23%
16			*O.sanum*			15			15	3.39%
Total				70	189	25	96	62	442	100.00%

^1^ PrJ = *Polygraphusrudis* from Jilong County; PrY = *P.rudis* from Yadong County; PrZ = *P.rudis* from Zeku County; PpZ = *P.poligraphus* from Zeku County; PpQ = *P.poligraphus* from Qilian County.

The dominant species in this study were *O.maixiuense*, *O.bicolor*, *L.yadongense* and *O.pseudobrevipilosi*, representing 20.8%, 18.1%, 17.4% and 13.4% of the ophiostomatalean isolates, respectively, while the other 12 species all had < 10% (Table 3). *Ophiostomamaixiuense* was first reported to be associated with *Dendroctonusmicans* infesting *P.crassifolia* on the north-eastern Qinghai-Tibet Plateau (Wang et al. 2021). This species was also obtained from *P.poligraphus* from the same host tree and sampling location. In addition, although several fungal associates of *P.rudis*, *I.schmutzenhoferi* and *I.stebbingi* have been isolated from *P.griffithii* in the Jilong and Yadong Counties on the southern Qinghai-Tibet Plateau, only *O.maixiuense* was shared (Table 3; Wang et al. (2024)). Therefore, this species may be widespread on the Qinghai-Tibet Plateau and its pathogenicity to host trees and association with bark beetles deserve further study. *Ophiostomabicolor* is frequently associated with bark beetles that harm spruce trees, such as some *Ips* and *Polygraphus* beetles in the Northern Hemisphere (Yamaoka et al. 1997; Kirisits 2004; Alamouti et al. 2007; Chang et al. 2019, 2020; Wang et al. 2021, 2024). It plays multiple roles in the association between beetles and spruce. Solheim (1988) found that it is weakly pathogenic to spruce trees, which may induce the host defence rather than deplete it (Liu et al. 2022). This is not necessarily beneficial during the early stages of insect vector attacks on trees (Mageroy et al. 2020). Conversely, although *O.bicolor* is not attractive to *I.typographus* (Kandasamy et al. 2019; Zhao et al. 2019), *I.nitidus* prefers to feed on *O.bicolor*-colonised substrates and may benefit from their aid in detoxification and improved ecological fitness (Wang et al. 2023). The mechanisms underlying the functional diversity traits in *O.bicolor* and their roles in tree–beetle–fungal interactions need to be further explored.

Comparisons of the fungal assemblages of bark beetles from the north-eastern and southern Qinghai-Tibet Plateau showed different patterns (30 vs. 12 fungal species), with only *O.maixiuense* being a shared species (Suppl. material 1: tables S2, S3), which may be due to biogeographic barriers and host species. On the north-eastern Qinghai-Tibet Plateau, there are 14, 21 and 14 fungal associates of *Dendroctonus*, *Ips* and *Polygraphus*, respectively (Yin et al. 2016, 2019, 2020; Chang et al. 2020; Wang et al. 2021, 2024). *Ophiostomaainoae*, *O.bicolor*, *O.nitidum*, *O.sanum* and *O.shangrilae* are shared by these three beetle genera, the latter three of which are currently found only on the Qinghai-Tibet Plateau, whereas the first two are thought to be widely distributed in the coniferous forests of China and are associated with a variety of bark beetles (Chang et al. 2019; Wang et al. 2024). Six fungal associates of *Polygraphus* were shared only with *Ips* and only two were shared with *Dendroctonus* (Suppl. material 1: table S2). This may be because of overlap in the niches of the first two genera of beetles. Furthermore, *Dendroctonus* mainly harms trunks below the DBH (diameter at breast height) of the host tree, which is not the preferred choice for *Polygraphus* and *Ips*. On the southern Qinghai-Tibet Plateau, although the straight-line distance between Jilong and Yadong Counties is not large, the fungal assemblages of bark beetles from the two Counties are divergent, with only one of the 12 species shared (Suppl. material 1: table S3). Interestingly, the fungal associations of different beetles at the two sites were highly coincident. Four of the six fungal associates of *P.rudis* are shared with *I.stebbingi*. Similarly, all four of the *P.rudis*’ fungal associates in Yadong County were also isolated from *I.schmutzenhoferi* by Wang et al. (2024). This suggests that the biogeographic barrier caused by the high mountain-and-gorge landform on the southern slopes of the Himalayas creates this fungal assemblage pattern of bark beetles, even though the host species are the same and geographical distances are not far.

Overall, this study deepens our understanding of the composition of ophiostomatoid fungi associated with bark beetles, especially *Polygraphus*, on the Qinghai-Tibet Plateau. The discovery of a large number of new fungal species and new tree-bark beetle-fungal associations has made it an urgent task to reveal their biological functions and ecological features.

## Supplementary Material

XML Treatment for Leptographium pseudojilongense

## ﻿Additional information

### Author ORCIDs

Zheng Wang https://orcid.org/0000-0003-0207-4321

Quan Lu https://orcid.org/0000-0002-6007-2677

### Data availability

All of the data that support the findings of this study are available in the main text or Supplementary Information.

## Supplementary materials

Supplementary material 1 Supplementary tables Data type rar Explanation note **table S1.** List of sampling information of *Polygraphus* bark beetles. **table S2.** Comparisons of fungal assemblages of bark beetles in the north-eastern Qinghai-Tibet Plateau. **table S3.** Comparisons of fungal assemblages of bark beetles in the southern Qinghai-Tibet Plateau. https://binary.pensoft.net/file/1165798This dataset is made available under the Open Database License (http://opendatacommons.org/licenses/odbl/1.0/). The Open Database License (ODbL) is a license agreement intended to allow users to freely share, modify, and use this Dataset while maintaining this same freedom for others, provided that the original source and author(s) are credited.Zheng Wang, Caixia Liu, Xiuyue Song, Yingjie Tie, Huimin Wang, Huixiang Liu, Quan Lu

Supplementary material 2 Supplementary figures Data type rar Explanation note **figure S1.** Phylogram of *Grosmanniapenicillata* complex (including Taxa 1–3) based on *tub2* sequence data. **figure S2.** Phylogram of *Grosmanniapenicillata* complex (including Taxa 1–3) based on *tef1-α* sequence data. **figure S3.** Phylogram of *Leptographiumlundbergii* complex (including Taxa 4–7) based on *tub2* sequence data. **figure S4.** Phylogram of *Leptographiumlundbergii* complex (including Taxa 4–7) based on *tef1-α* sequence data. **figure S5.** Phylogram of *Leptographiumolivaceum* complex (including Taxon 8) based on *tub2* sequence data. **figure S6.** Phylogram of *Leptographiumolivaceum* complex (including Taxon 8) based on *tef1-α* sequence data. **figure S7.** Phylogram of *Ophiostomaclavatum* complex (including Taxa 9–11) based on *tub2* sequence data. **figure S8.** Phylogram of *Ophiostomaclavatum* complex (including Taxa 9–11) based on *tef1-α* sequence data. **figure S9.** Phylogram of Group A (including Taxa 14–16) based on ITS sequence data. The ML bootstrap support values ≥ 70% and posterior probability values ≥ 0.9 are recorded at the nodes. T = ex-type isolates. **figure S10.** Phylogram of Group A (including Taxa 14–16) based on *tub2* sequence data. https://binary.pensoft.net/file/1165799This dataset is made available under the Open Database License (http://opendatacommons.org/licenses/odbl/1.0/). The Open Database License (ODbL) is a license agreement intended to allow users to freely share, modify, and use this Dataset while maintaining this same freedom for others, provided that the original source and author(s) are credited.Zheng Wang, Caixia Liu, Xiuyue Song, Yingjie Tie, Huimin Wang, Huixiang Liu, Quan Lu
